# Causes of death in patients with extranodal cancer of unknown primary: searching for the primary site

**DOI:** 10.1186/1471-2407-14-439

**Published:** 2014-06-14

**Authors:** Matias Riihimäki, Akseli Hemminki, Kristina Sundquist, Kari Hemminki

**Affiliations:** 1 Division of Molecular Genetic Epidemiology, German Cancer Research Centre (DKFZ), Heidelberg 69120, Germany; 2 Center for Primary Health Care Research, Lund University, Malmö, Sweden; 3 Cancer Gene Therapy Group, Transplantation Laboratory & Department of Pathology, Haartman Institute, University of Helsinki, Helsinki 00290, Finland; 4 Stanford Prevention Research Center, Stanford University School of Medicine, Palo Alto, California, USA

**Keywords:** Cancer of unknown primary, CUP, Cause of death

## Abstract

**Background:**

Cancer of unknown primary (CUP) is a fatal cancer, accounting for 3–5% of all diagnosed cancers. Finding the primary site is important for therapeutic choices and we believe that the organ which is designated as the cause of death may give clues about the primary site.

**Methods:**

A total of 20,570 patients with CUP were identified from the Swedish Family-Cancer Database. Causes of death – as reported in the death certificate - were investigated, analyzing reported metastatic sites and histological subtypes separately. Survival was compared with metastatic cancer with a known primary tumor.

**Results:**

An organ-specific cancer could be identified as a cause of death in approximately 60% of all CUP patients with adenocarcinoma or undifferentiated histology. In adenocarcinoma, lung cancer was the most frequent cause of death (20%), followed by pancreatic cancer (14%), and ovarian cancer (11%). Lung cancer was the most common cause of death in patients with CUP metastases diagnosed in the nervous system (69%), respiratory system (53%), and bone (47%), whereas ovarian cancer was the most common cause of death when CUP was diagnosed in the pelvis (47%) or the peritoneum (32%). In CUP diagnosed in the liver, liver and pancreatic cancers accounted for 26% and 22% of deaths, respectively. Also in squamous cell CUP, lung cancer was the most common cause of death (45%).

**Conclusions:**

According to the causes of death, the primary site appeared frequently to be either the organ where CUP metastases were diagnosed or an organ which may be traced through the known metastatic patterns of different cancer types.

## Background

Cancer of unknown primary (CUP) is a group of cancers, frequently cited to account for 3–5% of newly diagnosed cancers [1-5]. Despite its high incidence, very little is known about this challenging entity of cancers. Most patients with CUP have a dismal prognosis: survival estimates are often in the magnitude of a few months [6,7], although it may be longer in hospital-based studies with selected populations [8,9]. Especially, CUP with metastases in the liver has a poor prognosis, with patients only surviving a median of two months [8-12]. However, some favorable subsets have been described, such as neuroendocrine CUP, and CUP limited to lymph nodes [2,3,9,13]. CUP poses a challenge for the treating physician, because cancer treatment is typically planned based on the primary tumor; therefore, upon detection of a metastasis, a wide diagnostic workup is initiated to detect the primary site [2,3,9,14-17]. Despite recent advances in diagnostic modalities, including imaging techniques, expression profiling, and immunohistochemical methods, not all primary tumors are found, and some cancers remain cancers of unknown primary. Ultimately, in some cases the primary site may be detected by autopsy, and is then often situated in the lungs or gastro-intestinal system [13,14]. However, in some cases no primary is found, presumably reflecting the immunological eradication of the original tumor subsequent to shedding of metastases.

Although being a diverse group of cancers, some clinical features distinguish CUP [2,18]. The primary tumor has often regressed, or may even have disappeared. Furthermore, patients with CUP typically have metastases, and the disease is often characterized by resistance to chemotherapy. CUP often displays biological features, such as aneuploidy [1,3]. Furthermore, some metastasis-related genes are frequently overexpressed, e.g. c-Myc [1], MET [17], MMPs [19], and VEGF [18]. In family studies, CUP could be linked with several other cancers, e.g. liver, lung, pancreas, and ovaries [4,5]. Another approach has been to investigate the etiology of CUP utilizing death certificates of patients with CUP; indeed cancers of the lung, liver, pancreas, and ovaries are common causes of death after diagnosis of CUP [5,20]. In CUP limited to lymph nodes, lung cancer is the most common specified cancer in death certificates, especially in CUP detected in thoracic lymph nodes, which supports the notion that the primary cancer originates in the lung [21].

In the present study, we investigated the causes of death in extranodal CUP, depending on the location of CUP metastases at diagnosis. Different histological subtypes were analyzed separately (adenocarcinoma/undifferentiated cancer, squamous cell cancer, and melanoma). We allow that the tumor resulting in CUP diagnosis may in fact be different from the original primary tumor, since when CUP is diagnosed, the primary is by definition not known. However, our hypothesis is that the tumor causing death may in fact represent the original primary tumor which could not be originally detected with the available imaging methods. Nevertheless, the primary may have persisted and ultimately resulted in death. We assumed that the known metastatic pathways of primary cancers also apply to the metastatic spreading of CUP. To dissect our hypothesis, the survival kinetics of patients with CUP was compared with the survival of patients with known primaries.

## Methods

The dataset used in this study was obtained from the most recent update of the Swedish Family-Cancer Database (FCD), which is a national research database located at the Center for Primary Health Care Research, Malmö, Sweden. The FCD contains, e.g., cancer data from the Swedish Cancer Registry and death statistics from the Cause of Death Registry [22], and has been used in several population-based studies on CUP [4,5,9,11,12,21,23]. All cancer cases diagnosed from 1958 through the end of 2010 are included. The Swedish Cancer Registry, which is based on the compulsory notification of cancer cases and the completeness of cancer registration has been approximated to be over 90% [24].

CUP cases were identified by their ICD-codes (International Classification of Disease), as reported in the Cancer Register. The Cancer Register has used ICD-7 since 1958, ICD-9 since 1987, ICD-O/2 since 1993, and ICD-O/3 since 2005. All subsequent versions in the Cancer Register are also translated to older versions, enabling comparisons over time. ICD-9 coding, and later versions, allow identification of the site of CUP metastases. The Cause of Death Registry used ICD-9 coding between 1987 and 1996, and ICD-10 coding since 1997 [22]. Accordingly, the follow-up time was 1987 through 2010. SAS software was used for statistical analyses. Kaplan Meier plots were generated with PROC LIFETEST (SAS Version 9.3; SAS Institute, Cary, NC).

The locations of CUP metastases were identified by their ICD-9 code: respiratory system (195.1, 197.0-3), liver (197.7), peritoneum/retroperitoneum (195.2, 197.6), nervous system (198.3-4), bone (198.5), pelvis (195.3), other specified (195.0, 4, 5, 8, 9; 198.0-2, 6–9), and “unspecific cup/CUP C80” (199). ICD-9 and -10 codes 199 and C80 correspond to unspecified CUP location, both in the Cancer Registry and the Cause of Death Registry. Primary cancer sites were identified by their ICD-7 codes, and histological subtypes were identified by their WHO/HS/CANC/24.1 (pad) codes. Previous reports have shown that adenocarcinoma and undifferentiated histology are similar, thus these two histological types were analyzed together [9]. Unspecified and other histological subtypes than adenocarcinoma/undifferentiated, squamous cell, and melanoma were not included in the analyses.

## Results

A total of 28,419 CUP patients diagnosed between 1987 and 2010 were identified from the FCD. Of these, 26,811 (93%) died during the study period. Adenocarcinoma was the most common histology (14,947 cases [55.%]), followed by undifferentiated (4143 cases [15.5%]), squamous cell (773 cases [2.9%]), and melanoma (707 cases [2.6%]). Unspecified histology (5185 cases [19.3%]) and other specified histological subtypes (1,056 cases [3.9%]) were omitted from analyses.

In Table 1, the underlying causes of death in patients with CUP (adenocarcinoma/undifferentiated) are displayed, depending on the location of CUP metastases at diagnosis. Overall, of all specified cancer causes of death, lung cancer was the most common (20%), followed by pancreatic cancer (14%), and ovarian cancer (11%). Substantial differences could be noted in causes of death between different CUP sites. The most common specific CUP location was the liver. Of all specified cancer causes of death, the most common for CUP of liver were cancer of liver (26%), pancreas (22%), colorectum (13%), and biliary system (13%). In CUP of the peritoneum, the causes of death were different: ovarian cancer (32%), pancreatic cancer (16%), and colorectal cancer (12%). Lung cancer was the most frequently mentioned cause of death when CUP was diagnosed in the bone (47%), the nervous system (69%), and the respiratory system (53%), but not as common in CUP of the peritoneum (1%), and the pelvis (1%). Ovarian cancer was also a common cause of death when CUP was diagnosed in the pelvis (47%) and peritoneum (32%). The group “other cancer” was large in CUP of the nervous system and pelvis. This group constitutes e.g. brain tumors, which was a common death cause in CUP of the nervous system (23 cases, 8%), and female genital cancer, which was a common cause of death in CUP of pelvis (85 cases, 25%).

**Table 1:** Causes of death (from the Swedish cause of death registry) in patients with CUP (adenocarcinoma and undifferentiated cancer) depending on site in the Swedish cancer register

	**Location of CUP**																	
**Cause of death**	**Liver**		**Peritoneum**		**Respiratory system**		**Bone**		**Pelvis**		**Nervous system**		**Other specified**		**C80/199**		**All**	
**Lung**	189	8%	29	1%	558	**53%**	217	**47**%	3	1%	194	**69%**	184	**27%**	934	**24%**	2308	**20%**
**Pancreas**	551	22%	371	16%	31	3%	19	4%	4	1%	2	1%	66	10%	523	13%	1567	14%
**Ovaries**	25	1%	765	**32%**	33	3%	1	0%	160	**47%**	0	0%	34	5%	284	7%	1302	11%
**Colorectum**	330	13%	291	12%	17	2%	17	4%	26	8%	9	3%	82	12%	362	9%	1134	10%
**Liver**	649	**26%**	56	2%	13	1%	14	3%	2	1%	0	0%	10	1%	353	9%	1097	9%
**Biliary system**	310	13%	161	7%	7	1%	5	1%	0	0	0	0%	31	5%	285	7%	799	7%
**Ill-defined gastro-intestinal**	202	8%	154	6%	13	1%	12	3%	4	1%	1	0%	33	5%	219	6%	638	6%
**Mediastinum**	1	0%	13	1%	272	26%	3	1%	1	0%	4	1%	10	1%	114	3%	418	4%
**Urinary system**	28	1%	27	1%	22	2%	60	13%	12	4%	13	5%	51	7%	139	4%	352	3%
**Stomach**	50	2%	120	5%	12	1%	4	1%	3	1%	2	1%	38	6%	94	2%	323	3%
**Peritoneum**	5	0%	160	7%	5	0%	0	0%	10	3%	0	0%	6	1%	76	2%	262	2%
**Breast**	16	1%	6	0%	11	1%	24	5%	1	0%	3	1%	33	5%	70	2%	164	1%
**Prostate**	3	0%	4	0%	2	0%	34	7%	4	1%	6	2%	11	2%	76	2%	140	1%
**Other cancer**	100	4%	223	9%	58	6%	52	11%	108	32%	48	17%	92	14%	363	9%	1044	9%
**Any specified cancer**	2459	100%	2380	100%	1054	100%	462	100%	338	100%	282	100%	681	100%	3892	100%	11548	100%
**CUP C80**	1456		1456		313		398		122		106		287		2110		6248	
**Non-cancer**	175		175		122		85		41		32		107		557		1294	
**Total**	4090		4011		1489		945		501		420		1075		6559		18330	

CUP = Cancer of unknown primary. C80/199 = CUP with unspecified location. The most common cause of death is bolded.

**Figure 1:**
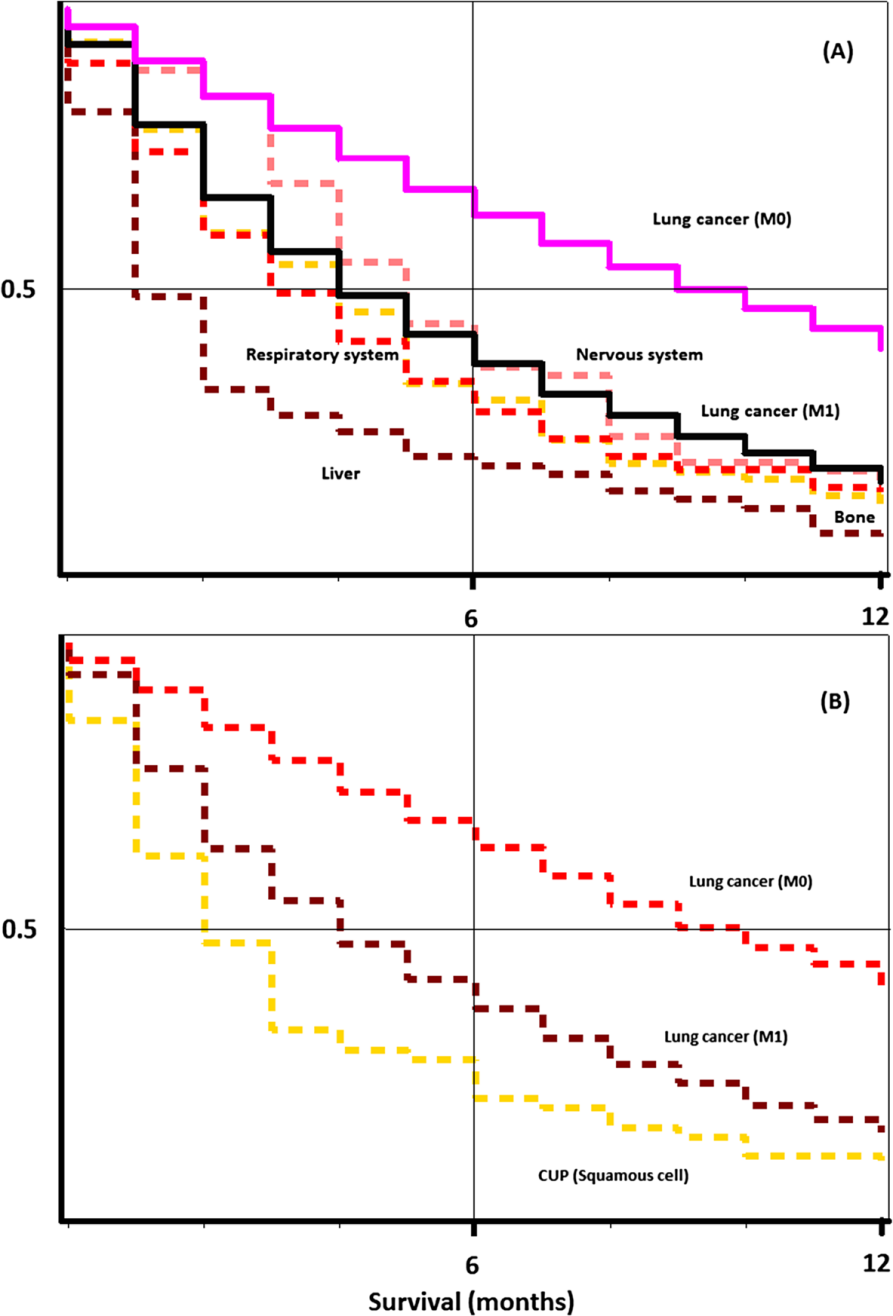
**Kaplan-Meier curves for metastatic (M1) and non-metastatic (M0) non-CUP lung cancer patients, compared with lung cancer deaths (according to the death certificate) of CUP patients with CUP diagnosed in specific locations (Nervous system, respiratory system, liver, bone).** The upper panel **(A)** includes adenocarcinoma only, and the lower panel **(B)** depicts squamous cell cancer.

**Figure 2:**
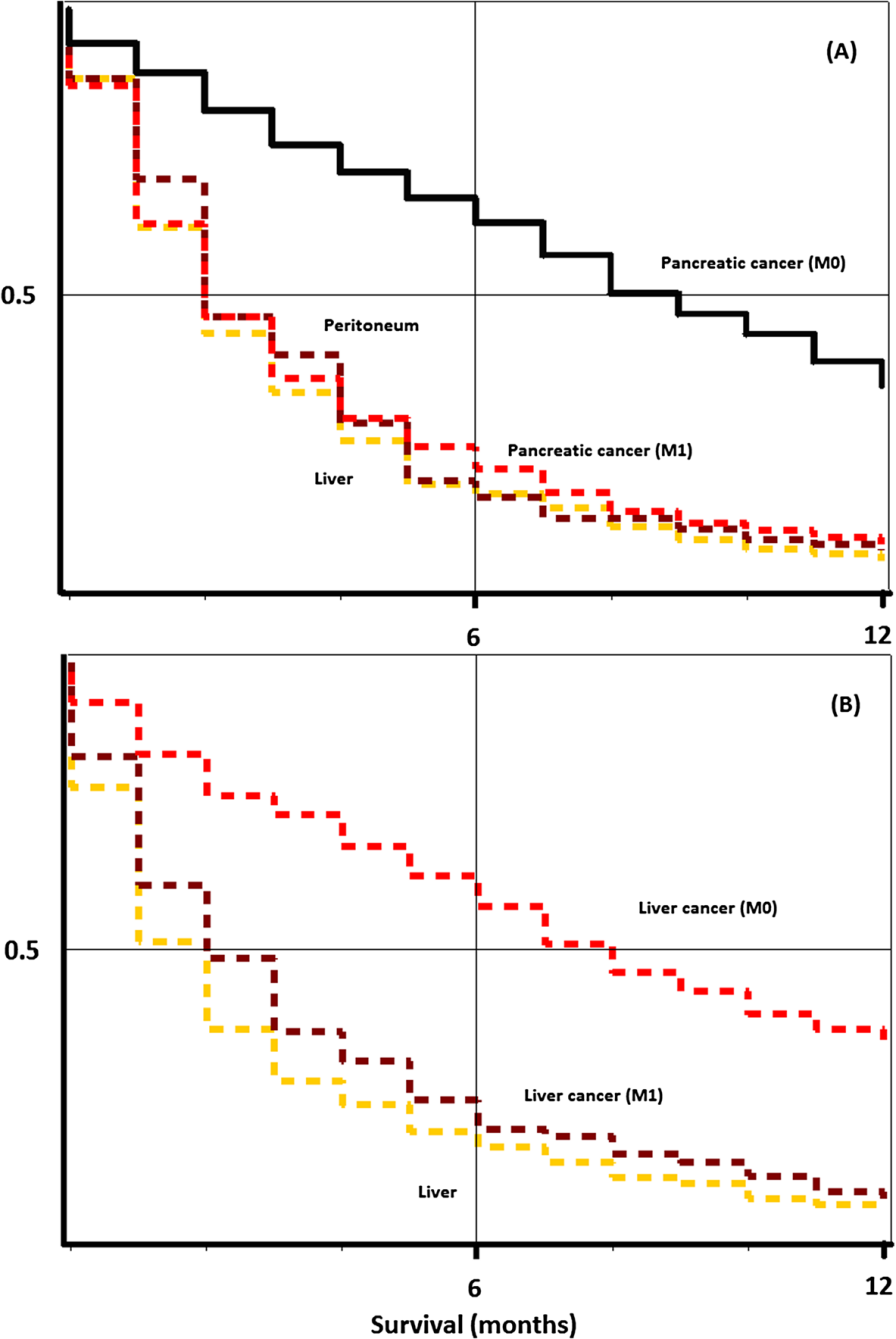
Kaplan-Meier curves for metastatic (M1) and non-metastatic (M0) non-CUP liver (A) and pancreatic (B) cancer patients, comparing to survival of patients with an initial diagnosis of CUP, eventually dying of liver or pancreatic cancer, according to their death certificates.

In Table 2, causes of death in patients with squamous cell CUP are displayed. Of all specified cancer causes of death, lung cancer (45%) was the most common. Lung cancer was the most common cause of death when CUP was diagnosed in the nervous system (90%), bone (77%), and respiratory system (62%). Upper aerodigestive tract cancer accounted for 32% of deaths in patients who had CUP diagnosed in the head. Notably, the proportion of non-cancer causes of death was substantially higher in squamous cell CUP than in adenocarcinoma/undifferentiated CUP. If the histological subtype of CUP was melanoma (707 cases, not displayed), the cause of death was melanoma in 577 (81.6%) of cases, other cancer than melanoma in 57 (8.1%), and unspecified CUP in 18 (2.5%) cases. 55 patients (7.8%) died from non-cancer causes.Survival in CUP was compared with survival in cancer with known primaries. In Figure 1, survival in CUP patients with lung cancer as the death cause was compared with primary lung cancer, metastatic (TNM stage M1) and non-metastatic (TNM stage M0) at diagnosis. The upper panel depicts patients with adenocarcinoma and undifferentiated histology. Four CUP sites were analyzed: liver, nervous system, bone, and respiratory system. Survival was similar in CUP and in stage M1 lung cancer, although CUP of liver featured the worst prognosis. In the lower panel, analysis was restricted to squamous cell histology. Survival in patients with CUP was slightly worse than in stage M1 lung cancer.In Figure 2, survival in CUP patients with liver (upper panel) and pancreatic cancer (lower panel) as cause of death was compared with metastatic (M1) and non-metastatic (M0) pancreatic cancer. Survival is very similar in both CUP of the liver and the peritoneum, compared with M1 pancreatic cancer. Liver cancer deaths in patients with CUP of the liver were compared with M1 and M0 liver cancer. Again, survival was very similar in both CUP and metastatic cancer.

**Table 2:** Causes of death (from the Swedish cause of death registry) in patients with CUP (Squamous cell cancer) in the Swedish cancer registry

	**Site of CUP**																			
**Cause of death**	**Head**		**Respiratory system**		**Liver**		**Peritoneum**		**Bone**		**Pelvis**		**Nervous system**		**Other specified**		**199/C80**		**All**	
**Lung**	8	24%	**37**	**62%**	**11**	**29%**	2	9%	**17**	**77%**	0	0%	**19**	**90%**	**15**	**38%**	**91**	**48%**	**200**	**45%**
**Upper aerodigestive**	**11**	**32%**	1	2%	1	3%	0	0%	0	0%	0	0%	0	0%	4	10%	15	8%	32	7%
**Mediastinum**	0	0%	16	27%	0	0%	0	0%	0	0%	0	0%	0	0%	0	0%	7	4%	23	5%
**Urinary system**	1	3%	0	0%	0	0%	2	9%	1	5%	**5**	**31%**	0	0%	4	10%	9	5%	22	5%
**Skin**	5	15%	0	0%	1	3%	0	0%	3	14%	0	0%	0	0%	3	8%	7	4%	19	4%
**Oesophagus**	1	3%	2	3%	0	0%	0	0%	0	0%	0	0%	1	5%	3	8%	10	5%	17	4%
**Liver**	0	0%	1	2%	10	26%	0	0%	0	0%	0	0%	0	0%	0	0%	6	3%	17	4%
**Pancreas**	0	0%	1	2%	5	13%	**3**	**13%**	1	5%	0	0%	0	0%	1	3%	4	2%	15	3%
**Biliary system**	0	0%	0	0%	6	16%	2	9%	0	0%	0	0%	0	0%	0	0%	5	3%	13	3%
**Other cancer**	8	24%	2	3%	4	11%	14	61%	0	0%	11	69%	1	5%	10	25%	34	18%	84	19%
**Any specified cancer**	34	100%	60	100%	38	100%	23	100%	22	100%	16	100%	21	100%	40	100%	188	100%	442	100%
**CUP C80**	24		10		27		23		15		13		6		17		77		212	
**Non-cancer**	21		7		1		2		3		5		3		8		69		119	
**Total**	80		77		66		48		40		34		30		65		334		773	

CUP = Cancer of unknown primary. C80/199 = CUP with unspecified location. The most common cause of death is bolded.

## Discussion

In the present study, we investigated causes of death in CUP patients. Distinct patterns could be seen in the causes of death depending on the location of CUP metastases at diagnosis. Overall, lung cancer was the most common cause of death when the initial site of CUP (adenocarcinoma/undifferentiated) was the nervous system (69%), respiratory system (53%), or bone (47%). On the other hand, ovarian cancer was a common cause after diagnosis of CUP in the pelvis (47%) and peritoneum (32%). We suggest that the cause of death, as determined in the death certificate, at least for some CUP patients point to the origin of primary tumor. For CUP diagnosed in the respiratory system lung and mediastinal cancers accounted for 79% of specified cancer deaths and survival curves were identical with primary metastatic lung cancer, which is compatible with the notion that the primary was a lung cancer that evaded detection during diagnostic workup. Similarly, for CUP located in the liver at diagnosis liver/pancreas/biliary system deaths accounted for 61% of all specific cancer deaths and survival kinetics were identical with metastatic liver cancer. Lung cancer deaths in patients whose CUP was located in the nervous system and bone is consistent with known metastatic pathways, as are ovarian cancer deaths with CUP locations of the peritoneum and pelvis. Thus we suggest that CUP location at diagnosis should direct a careful search of the primary to the likely sites defined here. This also has implications with regard to therapeutic choices.

Many of the patterns seen may be attributed to the known metastatic pathways [25,26]. For example, ovarian cancer frequently metastasizes within the pelvis and the peritoneum. Pancreatic cancer frequently metastasizes to the liver. Lung cancer often metastasizes within the respiratory organs, or to the nervous system and bone. Kidney cancer (included in “urinary system”) is also known to metastasize to the skeletal system. Considering the high incidence of bone metastases among breast and prostate cancer patients [27], it may be surprising to note that breast and prostate cancer are relatively uncommon causes of death in patients with CUP of bone, compared with lung cancer. There may be many reasons for this finding. The development of bone metastases is influenced by how long the patient lives with the tumor [27], and the primary tumor should be detected during that time. Also, because breast and prostate cancer are relatively easily detected by mammography and PSA tests, is seems reasonable to assume that many such tumors should be correctly diagnosed, and therefore not noted as CUP. Our results rely heavily on the accuracy of death certificates in Sweden. Generally, cause of death coding in Sweden is considered to be reliable, especially if the underlying cause of death is cancer [11,12,21]. Most cancer deaths occur in hospitals, and regardless of the location of death regulations specify that the issuer of the death certificate was either involved in the treatment of the patient, or profoundly educated themselves with the medical record. In unclear cases autopsy has been traditionally employed. Either way, the issuer of the death certificate has intimate knowledge of the patient’s medical history including the different phases of the malignancy.

Notifications of cancer to the Swedish Cancer Registry require two separate reports, one by a clinician and the other by a pathologist. CUP is reported if no primary tumor is found after adequate diagnostic work-up. If a primary site is found later, CUP diagnosis may be changed to the true primary [21]. Death registration is a completely independent process through the Causes of Death Register. The data are reported by a medically qualified death registrar who is usually well aware of the clinical course of the disease because for over 90% of cancer deaths examination at hospital prior to death was the basis on which the death certificate was issued [12]. CUP offers a unique opportunity to monitor the sequence of events from the hidden primaries to fatal organ metastases because death certificates give the site of organ metastasis as the cause of death even if histopathology indicates CUP. This practice deviates from that of any other cancer for which the primary cancer is given as the cause of death. Autopsy data are not used to change CUP diagnosis and nor would they be helpful because autopsy rates in Sweden have steadily declined, from 25% in 1987 to 7% in 2010 (women), and from 33% to 16% (men) [28]. Furthermore, the rate is lower in cancer patients and elderly. Previous family studies have shown that the cause of death in patients with CUP often is the same that is diagnosed in a family member [4,5].

Two classic theories are often used to explain the metastatic process: the seed and soil hypothesis, and the anatomical/mechanical hypothesis [29]. The seed and soil hypothesis stipulates that the interaction between the tumor cell (“seed”) and the target organ (“soil”) is important for the development of metastases. This theory partly explains why some malignancies tend to metastasize to the same organ where the tumor originated, or to selected target organs, e.g. breast cancer to the other breast or to the brain, while prostate cancer is often multifocal locally or metastatic to the bone. Also, the anatomical/mechanical hypothesis has shown merit in explaining metastatic patterns, and can be presumed to be important in CUP. Due to the portal venous system, it is natural that the primary cancer in CUP of liver may often be found within the gastro-intestinal system. In nodal CUP, anatomical factors may also play an important role [21]. In CUP of lymph nodes in the head, neck, or thoracic region, the most common cause of death was lung cancer, whereas in axillary lymph node CUP, it was breast cancer. Although the present results imply that a primary tumor may be found in patients with CUP, it is important to remember that initiating excessive diagnostic maneuvers is not necessarily the best strategy for these patients.

Although the primary site in CUP may be explained through the metastatic patterns of common cancers, some evidence points out that the primary tumor in CUP might in fact reside in the same organ as CUP, or even be the primary tumor itself. Different explanations supporting this view have been presented in previous studies. If the CUP in fact is the primary cancer, the tumor may have undergone massive phenotypical changes, seeming to be metastatic tissue, whereby it is not diagnosed as a primary tumor [5]. Alternatively, if the hidden primary tumor in CUP has resided in the same organ as the metastasis, it may have regressed to an undetectable size, or even have been eradicated by immune surveillance [2,18]. In practice, this has manifested in two ways in previous epidemiological research: 1) the cause of death in many patients with CUP may frequently be scored as cancer of the same organ where CUP was detected [12,20], and 2) that CUP confers an increased risk of a second cancer diagnosis at some locations, including the lung, ovaries, and kidney, all of them common primary sites for metastases [23].

## Conclusions

We have investigated the causes of death in patients with CUP with the aim to explore the origins of this challenging disease. In approximately 60% of CUP patients, an organ-specific tumor was listed as the underlying cause of death, lung cancer being the most common. When CUP was localized to the nervous system, respiratory system and bone, the primaries should be preferentially searched in the lung. In medicine, therapy follows diagnosis and thus even if primaries are not found, the aforementioned biological notions have relevance for selecting a therapy for each patient. When CUP is diagnosed in the peritoneum or pelvis., even in the primary site cannot be identified, the initiation therapy targeting ovarian cancer could be considered [2]. Although we provide some insight to the conundrum CUP, further etiological research is motivated. Many patients with CUP are however elderly, and one must weigh the possible harmful effects of a prolonged diagnostic workup in these patients, especially if therapy is unlikely to result from a more accurate diagnosis [30]. However, in modern oncology, an increasing number of options are available to oncologists and thus the possibility of optimal diagnosis should not be overlooked, as some effective therapies can be administered with low toxicity.

### Ethical approval

This study was approved by the ethical committee at Lund University, Sweden.

## Competing interests

The authors declare that they have no competing interests.

## Authors’ contributions

KS provided the data. MR and KH designed the study. MR, AH, KS, and KH interpreted the results. MR drafted the manuscript. All authors helped write and critically reviewed the manuscript. All authors read and approved the final manuscript.

## Pre-publication history

The pre-publication history for this paper can be accessed here:

http://www.biomedcentral.com/1471-2407/14/439/prepub
